# The heterogeneity of store-operated calcium entry in melanoma

**DOI:** 10.1007/s11427-016-5087-5

**Published:** 2016-07-14

**Authors:** Robert Hooper, M. Raza Zaidi, Jonathan Soboloff

**Affiliations:** 1 Fels Institute for Cancer Research and Molecular Biology, Lewis Katz School of Medicine at Temple University, Philadelphia 19140, USA; 2 Department of Medical Genetics & Molecular Biochemistry, Lewis Katz School of Medicine at Temple University, Philadelphia 19140, USA

**Keywords:** melanoma, STIM1, Orai1, metastasis

## Abstract

Calcium is a key regulator of many physiological processes that are perturbed in cancer, such as migration, proliferation and apoptosis. The proteins STIM and Orai mediate store-operated calcium entry (SOCE), the main pathway for calcium entry in non-excitable cells. Changes in the expression and function of STIM and Orai have been found in a range of cancer types and thus implicated in disease progression. Here we discuss the role of STIM, Orai and the SOCE pathway in the progression of melanoma and explore how the heterogeneous nature of melanoma may explain the lack of consensus in the field regarding the role of SOCE in the progression of this disease.

## INTRODUCTION

Ultraviolet (UV) radiation can lead to DNA damage by causing adjacent thymine base pairs to form pyrimidine dimers (Goodsell, 2001). To protect against such damage, melanocyte cells, located in the basal layer of the epidermis, produce the UV absorbing pigment melanin for export to keratinocytes, the predominant cell of the skin (Raposo and Marks, 2007). Fair-skinned individuals, however, with low levels of skin melanin, are particularly susceptible to UV-induced DNA damage, the primary risk factor in developing skin cell cancers. Malignant melanoma is one such cancer, originating from the melanocyte cell lineage (Schadendorf et al., 2015). While in the radial growth phase in the skin, malignant melanoma is easily treatable by surgical excision of the cutaneous lesions. However, once melanoma enters the vertical growth phase, the cells infiltrate the dermis and metastasize, 5-year survival rate falls to 15% (http://www.cancer.org/research/cancerfactsfigures/cancerfactsfigures/cancer-facts-figures-2013), making this a particularly deadly disease. Understanding how and why melanoma transitions to a metastatic state is therefore key to preventing disease progression and ultimately improving patient prognosis.

## THE STORE-OPERATED CALCIUM ENTRY (SOCE) PATHWAY

**Figure 1:**
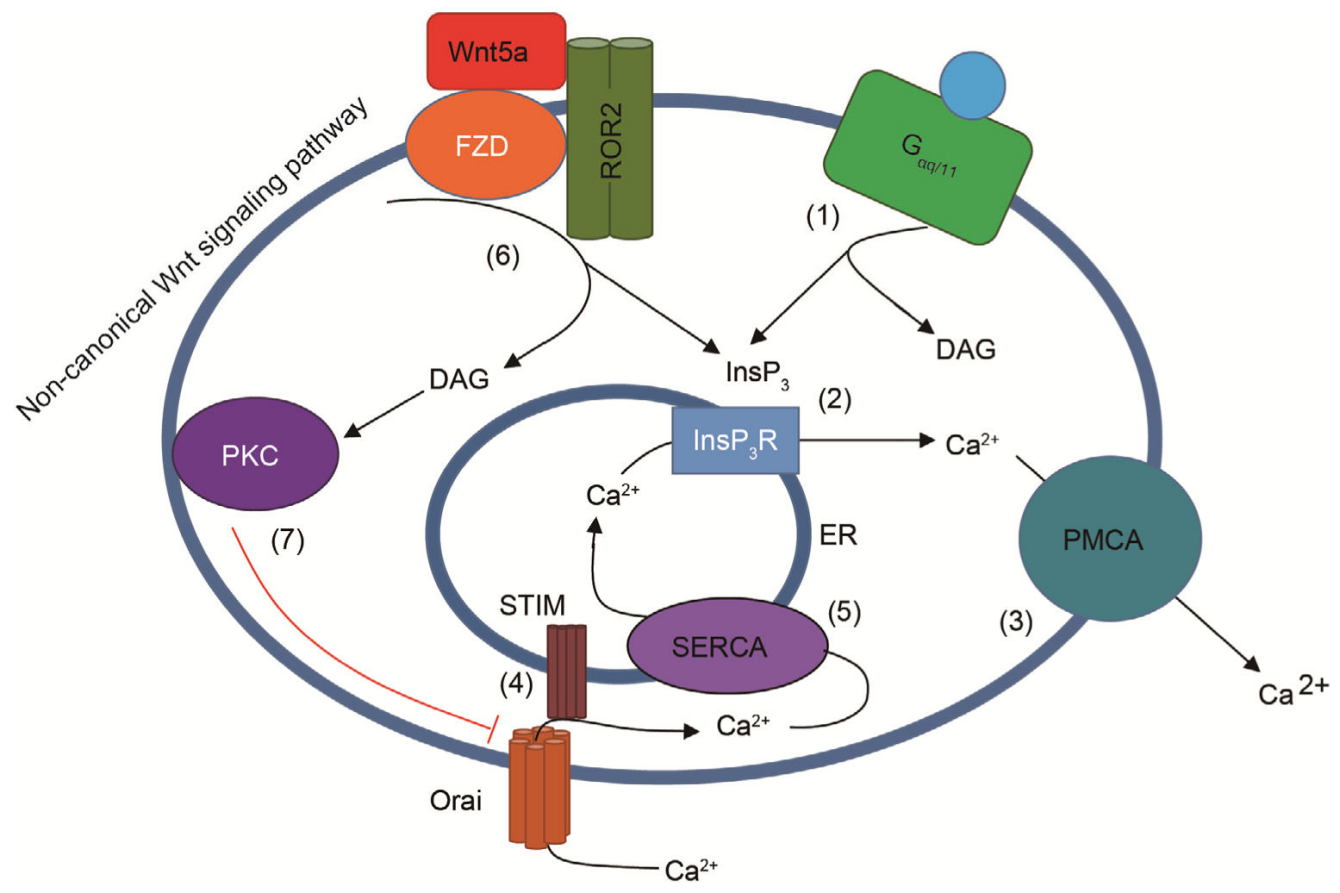
Wnt5a signals to inactivate Orai1. (1) Upon activation of phospholipase C (PLC)-coupled plasma membrane receptors (such as a G_αq/11_ coupled GPCR), phosphatidylinositol 4,5-bisphosphate (PIP_2_) is hydrolyzed to diacyl glycerol (DAG) and inositol 1,4,5-trisphosphate (InsP_3_). (2) InsP_3_ binds InsP_3_ receptors leading to Ca^2+^ release from the ER and (3) extrusion from the cell by the plasma membrane ATPase (PMCA). (4) As luminal ER Ca^2+^ concentration falls, Ca^2+^ dissociates from the EF-hands of STIM1 leading it to oligomerize and cluster at ER-PM junctions where it can gate Orai1 channels and facilitate store-operated calcium entry (SOCE) and (5) reuptake of Ca^2+^ into the ER via the sarco/endoplasmic reticulum Ca^2+^-ATPase (SERCA) pump. In melanoma cells, (6) Wnt5a activates the co-receptors *Frizzled* (*Fzd*) and receptor tyrosine kinase-like orphan receptor 2 (ROR2), part of the non-canonical Wnt signaling pathway, leading to the generation of DAG and InsP_3_. DAG activates PKC, which is known to phosphorylate Orai1 (7), leading to channel inactivation. Invasive melanoma cells with high levels of Wnt5a therefore have attenuated SOCE.

Calcium (Ca^2+^), a ubiquitous signaling ion and intracellular second messenger, is critical to a plethora of physiological processes, many of which are perturbed in cancer such as cell migration, proliferation and apoptosis (Berridge and Irvine, 1984). The primary store of intracellular Ca^2+^ is the endoplasmic reticulum (ER) with a luminal Ca^2+^ concentration of ~100 μmol L^−1^, approximately 1000-fold higher than the cytoplasm. Upon agonist activation of phospholipase C (PLC)-coupled cell surface receptors (such as G_αq/11_ coupled G-protein coupled receptors), phosphatidylinositol 4,5-bisphosphate (PIP_2_) is hydrolyzed to diacyl glycerol (DAG) and inositol 1,4,5-trisphosphate (InsP_3_), the latter of which acts upon ER-resident InsP_3_ receptors to release Ca^2+^ from the ER lumen (Figure 1). Ca^2+^ signals are finitely controlled by the amplitude, location, duration and frequency of Ca^2+^ release, the so called “Ca^2+^ signature” (Berridge et al., 2000) that governs downstream cellular response. Ca^2+^ is then extruded from the cell via the plasma membrane Ca^2+^ ATPase (PMCA) (Carafoli, 1991). As Ca^2+^ is released, its concentration in the ER lumen decreases leading to Ca^2+^ dissociation from the luminal EF-hands of ER-resident STIM proteins (Soboloff et al., 2012). Loss of Ca^2+^ binding leads to a conformational change in STIM, causing it to oligomerize and cluster at ER-plasma membrane (PM) junctions where the carboxy-terminus of the protein extends to interact with and activate PM Orai channels, gating Ca^2+^ influx. This process is termed store-operated Ca^2+^ entry (SOCE). There are two isoforms of STIM proteins and three Orai family members with STIM1 and Orai1 being the most extensively studied of these. Ca^2+^ is then taken up into the ER via the sarco/endoplasmic reticulum ATPase (SERCA) pump, refilling the intracellular store. Multiple other proteins are also integral to regulating Ca^2+^ entry and signaling, TRP channels, activated by a diverse array of stimulants (Venkatachalam and Montell, 2007), can mediate Ca^2+^ entry into a cell, independent of ER Ca^2+^ content, while Ca^2+^ is also pumped into mitochondria, the Golgi and endolysosomes within the cell (Xu et al., 2015).

## A ROLE FOR SOCE IN CANCER PROGRESSION

STIM1- and Orai1-generated Ca^2+^ signals have been implicated in cell migration and focal adhesion turnover in both normal cell function and disease models (Pan and Ma, 2015). Indeed, metastasis involves a loss of cell adhesion from the primary tumor, cell migration through extracellular matrix, adhesion to a secondary site then cell proliferation. Orai1 was shown to regulate focal adhesion formation at the leading edge of migrating HEK293 cells, while STIM1, perhaps independently from the process of SOCE, contributed to disassembly of focal adhesions at the rear of cells (Schafer et al., 2012). Broadly, knockdown of either of the primary SOCE components leads to a decrease in cell migration velocity. Indeed, migrating endothelial cells stimulated by chemoattractants, exhibit a front-to-rear Ca^2+^ gradient, with STIM/Orai-dependent Ca^2+^ pulses at the leading edge of the cell promoting the focal adhesion formation at the site of stimulation, thus driving directional movement (Tsai et al. 2014). STIM1 and Orai1 have also been demonstrated to play a role in cell migration and invasion in numerous cancers. Knockdown of either protein or pharmacological inhibition of SOCE was shown to increase focal adhesions in human MDA-MB-231 breast cancer cells and thus decrease tumor metastasis in a mouse model of breast cancer progression (Yang et al., 2009). Similarly, high levels of STIM1 were positively correlated to tumor invasion and metastasis in colorectal cancer, where endogenous levels were found to be upregulated in diseased tissue (Wang et al., 2015). STIM1 was found to drive migration by promotion of COX-2 expression and subsequent production of prostaglandin E2 in colorectal cancer cells. In brain tumor glioblastoma multiforme (GBM) cells, Orai1 levels were found to be higher than in human primary astrocytes, with an associated increase in the magnitude of SOCE in these cells (Motiani et al., 2013). Furthermore, knockdown of STIM1 and Orai1 led to a decrease in the ability of GBM cells to invade but with little effect on proliferation. Likewise, STIM1 was expressed at higher levels in gastric cancer cells than normal tissue, and again STIM1 knockdown inhibited cell migration and invasion (Xu et al., 2016). Therefore, in a variety of cancer types, both STIM1 and Orai1 were found to be upregulated and positively correlated to metastasis.

Conversely, in A459 lung cancer cells, overexpression of Orai1 or the use of pharmacological SOCE antagonists inhibited EGF-mediated cell proliferation by placing cells in G0/G1 cell cycle arrest (Hou et al., 2011). In human prostate cancer cells, downregulation of Orai1 and decreased SOCE was correlated to an apoptosis-resistant phenotype, suggesting that loss of SOCE could contribute to the uncontrolled proliferation of cancer cells (Flourakis et al., 2010). SOCE may therefore play differing roles in cancer progression depending on cell type or be locally altered in ways that promote migration and metastasis through control of focal adhesion turnover.

## WNT5A-MEDIATED ORAI1 INHIBITION IS CORRELATED TO MELANOMA INVASIVENESS

**Figure 2:**
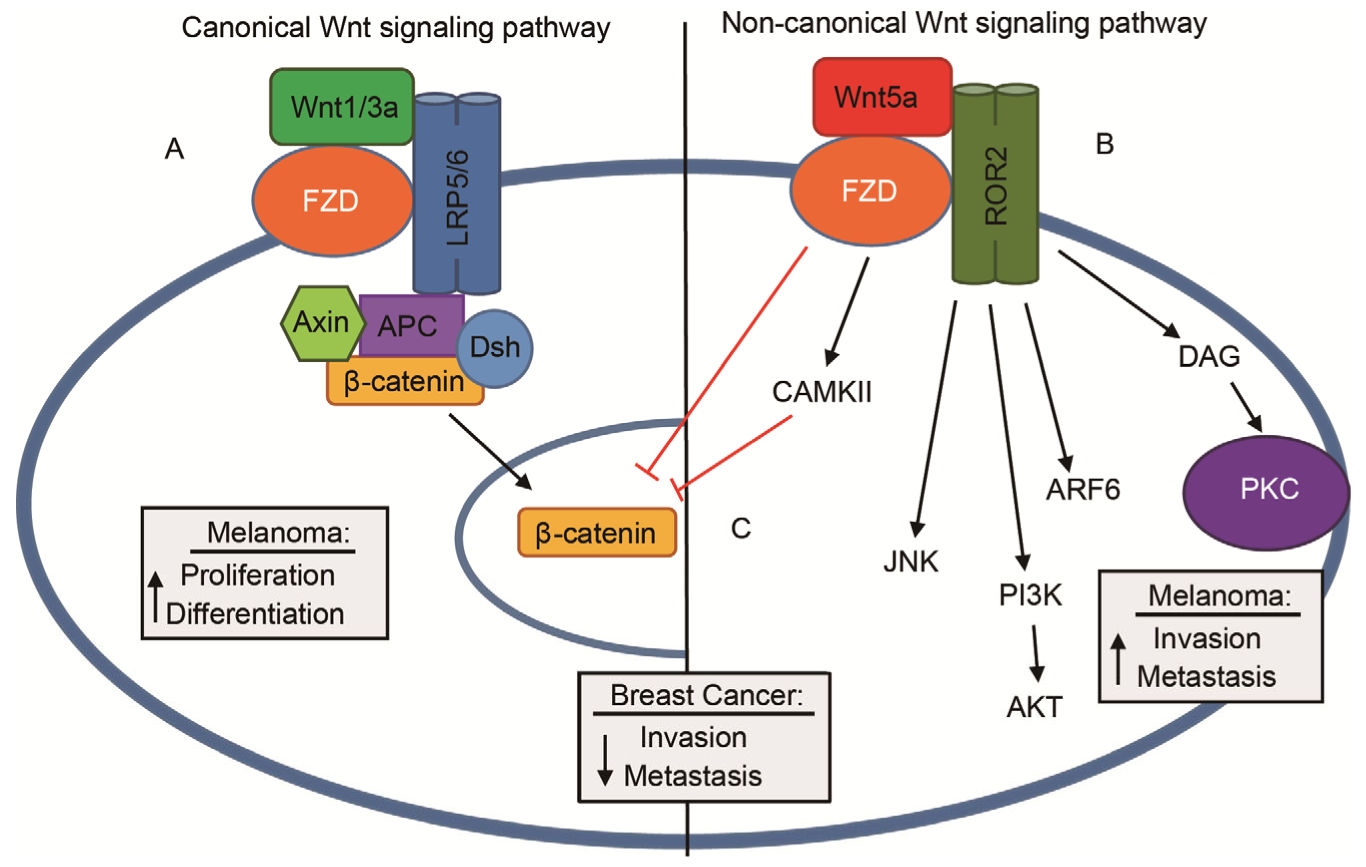
The canonical and non-canonical Wnt pathways have differing effects in cancer. A, Wnt1/3 binds the G-protein coupled receptor Fzd and the tyrosine kinase receptors LRP5/6 to initiate signaling through the canonical signaling pathway. The resulting signaling complex promotes β-catenin translocation to the nucleus and thus drives the radial growth phase of melanoma, whereby cells differentiate and proliferate on the dermis. B, An increase in Wnt5a activates the non-canonical Wnt signaling pathway through Fzd and the tyrosine kinase ROR2. Downstream effectors such as Arf6, Akt, Jnk and PKC drive a transition to the vertical growth phase, whereby melanoma invade through the dermis and metastasize. C, Conversely, in breast cancer cells, Wnt5a signaling though the non-canonical pathway activates CAMKII to facilitate β-catenin degradation, preventing the transcription of genes that promote metastasis and invasion.

We recently found that in patient-derived melanoma cell lines, those that were categorized as high in levels of Wnt5a and thus of an invasive phenotype, had dramatically less SOCE than non-invasive melanoma lines (Hooper et al., 2015). Wnt5a binds to the G-protein coupled receptor Fzd and the tyrosine kinase receptor ROR2 to mediate signaling. In melanoma, as well as gastric cancer and pancreatic adenocarcinoma, the non-canonical WNT pathway is engaged, independent of β-catenin, to activate downstream effectors such as PKC, Akt and Jnk and promote metastasis (Webster and Weeraratna, 2013; Zhu et al., 2014) (Figure 2). Conversely, in breast, thyroid, colon and hematopoietic cancers, increased Wnt5a expression can decrease metastasis. In these cancers, Wnt5a still binds to Fzd and ROR2, but receptor engagement instead inhibits the canonical Wnt-signaling pathway to promote β-catenin degradation and prevents the transcription of genes involved in tumor progression (Figure 2) (Zhu et al., 2014).

We observed that knocking down Wnt5a in invasive melanoma cell lines increased SOCE, while conversely overexpression of Wnt5a in non-invasive cells diminished SOCE to levels recorded in invasive cells (Hooper et al., 2015). This demonstrated a direct link between Wnt5a levels and SOCE in melanoma cells. The lack of SOCE in invasive cells was not however, due to any loss of SOCE components since expression of STIM and Orai proteins showed no correlation with invasiveness. Further, no defects in the localization or ability of these proteins to interact was observed, as evidenced by FRET studies. Finally, overexpression of STIM1 and Orai1 failed to restore SOCE in any of the Wnt5a-expressing invasive melanoma cell lines examined (Hooper et al., 2015).

It is known that when Wnt5a is upregulated, protein kinase C (PKC) activity is elevated (Dissanayake and Weeraratna, 2008). We found this elevated PKC activity was apparently responsible for the reduction in SOCE due to Orai1 phosphorylation, a process previously demonstrated to negatively regulate the channel *in vitro* (Kawasaki et al., 2010). Hence, use of the PKC inhibitor gö6983 or expression of an Orai1 protein insensitive to PKC-phosphorylation (Orai1^S27/30A^) led to SOCE restoration in invasive cells, but had no effect (relative to Orai1^WT^ for the latter) in non-invasive cells. Furthermore, both Wnt5a knockdown or PKC inhibition decreased the invasive potential of melanoma *in vitro*. Therefore an inverse relationship exists between Wnt5a levels/PKC activity and SOCE via Orai1 phosphorylation (Figure 1). It remains to be elucidated whether the loss of SOCE itself promotes invasion by altering Ca^2+^ dynamics or whether loss of SOCE is a byproduct of cells changing to an invasive phenotype.

## DIFFERING ROLES OF SOCE IN MELANOMA PROGRESSION

Interestingly, work by other groups studying melanoma cell lines with different characteristics has tended to support an alternative model in which melanoma invasiveness is positively correlated with SOCE, consistent with findings in other cancer types (Vashisht et al., 2015). One of the first reports on the role of STIM and Orai in melanoma examined mouse B16BL6 cells, where SOCE was shown to be enhanced in malignant cells with downstream activation of protein kinase B/Akt (Feldman et al., 2010). The authors found that while there were no differences in STIM1 or Orai1 expression between malignant and non-malignant cells, Orai1 likely remained open longer to increase influx and this elevated Ca^2+^ was buffered by the mitochondria. Notably, inhibiting mitochondrial Ca^2+^ uptake could decrease SOCE, suggesting a level of cross-talk between the two processes, with loss of SOCE leading to loss of PKB activation, ablated cell growth and increased cell susceptibility to apoptosis.

More recently, Stanisz et al. reported SOCE in both primary (SK-MEL-28) and metastatic (SK-MEL-5, WM3734) melanoma cells, although either pharmacological SOCE inhibition or knockdown of Orai1 and STIM2 resulted in faster growth, linked to malignancy (Stanisz et al., 2014). However, due to the commonly observed inverse relationship between proliferation and invasiveness (Liotta and Stetler-Stevenson, 1991), this fast growth leads to decreased invasive potential not dissimilar from the findings of our study (Hooper et al., 2015). In contrast, another group found that SOCE was actually enhanced in metastatic melanoma compared to non-metastatic primary melanoma cells and melanocytes (Umemura et al., 2014). Notably, unlike the melanoma lines characterized in (Hooper et al., 2015; Feldman et al., 2010; Stanisz et al., 2014), STIM1 and Orai1 were found to be highly expressed in melanoma, but no expression of their homologs was observed (Hooper et al., 2015; Feldman et al., 2010; Stanisz et al., 2014). Umemura et al. further found that suppression of SOCE by knockdown of STIM1 or Orai1 or use of the pharmacological SOCE inhibitor YM58483, drastically decreased cell migration in a Boyden chamber assay and decreased lung metastatic colonization post tail-vein injection (Umemura et al., 2014). These SOCE-mediated effects were attributed to activation of the extracellular-signal-regulated kinase (ERK) pathway and could be blocked by inhibitors of calmodulin kinase II (CaMKII) or Raf-1.

A role for STIM1 and Orai1-mediated Ca^2+^ oscillations was further demonstrated in the context of cell invadopodium assembly and extracellular matrix (ECM) degradation in models of melanoma metastasis (Sun et al., 2014). Ca^2+^ oscillations were also shown to be necessary for Src activation in melanoma cells, which recruits cortactin and the adaptor protein TKS5 to promote actin assembly and the formation of invadopodia structures (Sun et al., 2014). Further, Orai1-mediated signaling was found to be integral for trafficking MT1-matrix metalloproteinase (MMP) to the plasma membrane, where it facilities ECM degradation and promotes cell invasion. STIM1 and Orai1-mediated Ca^2+^ signals were thus shown to be implicated in processes necessary to metastasis. Indeed, knockdown of STIM1 inhibited melanoma lung metastasis in a mouse xenograft model (Sun et al., 2014).

## THE HETEROGENEITY OF MELANOMA: IMPLICATIONS FOR CA^2+^ SIGNALING

Melanoma is a highly heterogeneous disease, driven by a range of oncogenic proteins, which hampers effective treatment upon metastasis (Schadendorf et al., 2015). Our work focused on cells that were characterized as invasive based on Wnt5a expression and these cells showed greatly attenuated SOCE (Hooper et al., 2015). It is likely that other groups utilized invasive cells that had no differences in Wnt5a, indeed Wnt5a-positive invasive cells may represent a subset of metastatic melanomas and may require drastically different treatment strategies from invasive cells that aren’t driven by Wnt5a. In one study, Wnt5a protein and transcript levels were found to be upregulated in cell lines and patient samples that were resistant to BRAF-inhibitor (Anastas et al., 2014). Indeed, based on the publications discussed here, it could be envisaged that SOCE inhibition and SOCE enhancement may both represent valid treatment strategies tailored to different melanoma types, with the goal of “normalizing” SOCE in invasive, metastatic cells. Also it should be noted that, although we observe greatly attenuated SOCE in invasive cells compared to non-invasive, these cells still have some limited Ca^2+^ entry. Stanisz et al. show SOCE in SK-MEL-5 cells that has a peak amplitude similar to that of the Tg-induced ER Ca^2+^ blockade, while the SOCE observed in their WM3734 cells is almost 4-fold greater compared to the Tg-response (using control siRNA) (Stanisz et al., 2014), suggesting some dynamic range.

It is, perhaps, not surprising that melanoma cells with different genetic backgrounds exhibit differential dependence on SOCE, however, the interdependence of these phenomena has not been directly assessed. Further, the extent to which SOCE affects the invasive phenotype in these different genetic backgrounds remains unclear. Hence, a comprehensive screen of available melanoma cell lines and primary melanomas may provide new insight into both the regulation of SOCE in melanoma and how SOCE relates to the invasive features of this disease.

The expression and function of other Ca^2+^-permeable ion channels may also influence the dependence of melanoma on SOCE. Hence, transient receptor potential melastatin 1 (TRPM1), a member of the TRP super-family of ion channels, is expressed in melanocytes, where it is integral to melanin synthesis. Interestingly, loss of TRPM1 has been described as a marker of melanoma aggressiveness and tumor thickness (Miller et al., 2004) although how this affects Ca^2+^ homeostasis has not been investigated, nor has the influence of TRPM1 on SOCE in invasive melanoma been determined. Another member of the TRPM family, TRPM8, was shown to be functionally expressed in melanoma cells with sustained Ca^2+^ influx mediated by the channel agonist menthol leading to decreased viability of cells (Yamamura et al., 2008), although TRPM8 is also involved in normal melanocyte physiology so the extent to which this phenomenon is specific to melanoma is not clear.

Given the intimate relationship between Ca^2+^ signals and migration, investigations into how SOCE and other Ca^2+^ signaling pathways are altered in a range of melanoma cells may provide an invaluable guide for future therapies targeting Ca^2+^ homeostasis as a way to modulate melanoma metastasis.
